# Decolonization and Pathogen Reduction Approaches to Prevent Antimicrobial Resistance and Healthcare-Associated Infections

**DOI:** 10.3201/eid3006.231338

**Published:** 2024-06

**Authors:** Mihnea R. Mangalea, Alison Laufer Halpin, Melia Haile, Christopher A. Elkins, L. Clifford McDonald

**Affiliations:** Centers for Disease Control and Prevention, Atlanta, Georgia, USA (M.R. Mangalea, A.L. Halpin, M. Haile, C.A. Elkins, L.C. McDonald);; United States Public Health Service, Rockville, Maryland, USA (A.L. Halpin)

**Keywords:** Pathogen reduction, decolonization, healthcare-associated infection, bacteria, antimicrobial resistance, transmission prevention, human microbiome, patient safety

## Abstract

Antimicrobial resistance in healthcare-associated bacterial pathogens and the infections they cause are major public health threats affecting nearly all healthcare facilities. Antimicrobial-resistant bacterial infections can occur when colonizing pathogenic bacteria that normally make up a small fraction of the human microbiota increase in number in response to clinical perturbations. Such infections are especially likely when pathogens are resistant to the collateral effects of antimicrobial agents that disrupt the human microbiome, resulting in loss of colonization resistance, a key host defense. Pathogen reduction is an emerging strategy to prevent transmission of, and infection with, antimicrobial-resistant healthcare-associated pathogens. We describe the basis for pathogen reduction as an overall prevention strategy, the evidence for its effectiveness, and the role of the human microbiome in colonization resistance that also reduces the risk for infection once colonized. In addition, we explore ideal attributes of current and future pathogen-reducing approaches.

Interrupting transmission and infection caused by multidrug-resistant organisms (MDROs) in healthcare settings can help mitigate the global antimicrobial resistance (AMR) crisis, reduce illness and death, increase patient safety, and extend the usefulness of currently available antimicrobial medications. Past progress in this area showed infection prevention and control (IPC) strategies, along with antibiotic stewardship, saved lives and reduced unnecessary antibiotic use. The Centers for Disease Control and Prevention (CDC) reported that, from 2013 to 2019, deaths from AMR infections decreased by 18% overall and by 28% in hospitals (*1*). Despite that progress, during the COVID-19 pandemic, resistant healthcare-associated infections (HAIs) in the United States increased by 15%, possibly because of pandemic-related interruption of comprehensive prevention practices and antibiotic stewardship (*2*). Bacterial AMR remains a leading health issue nationally; >2.8 million persons are infected annually in the United States (*1*), and >4.95 million AMR-associated deaths were reported worldwide in 2019 (*3*).

Many HAIs are preceded by colonization with the infecting pathogen; colonized patients’ subsequent infection risk is increased by invasive devices, surgery, and receipt of antibiotics for prophylaxis or treatment for an unrelated infection (*4*–*7*). Pathogens that cause HAIs can be transmitted through direct or indirect contact from both asymptomatically colonized and symptomatically infected patients. Transmission to other persons at risk for colonization, infection, or both is especially notable because many risk factors for colonization overlap with those for infection. Newly colonized or infected persons then become potential secondary reservoirs for transmission. Hence, reducing pathogen burden in colonized patients could reduce not only risk to the original colonized person but also risk to the larger healthcare population. Here, we discuss current strategies for pathogen reduction that decolonize to various extents, evidence for effectiveness in preventing infections, potential future therapies leveraging the colonization resistance afforded by the microbiome, and outstanding needs in this area to promote infection prevention and patient safety.

## Role of Colonization in Pathogenesis

The human body is perpetually colonized by microorganisms (i.e., microbiota), along with those organisms’ associated metabolites and immediate environment (i.e., microbiome), which greatly influence human health and well-being. Most abundant in the intestines, mouth, skin, nose, and vagina, bacterial association with a human host can be transient or sequentially develop into life-long colonization (Table). Colonizing bacteria can be integrated as symbionts or pathobionts of the microbiome; pathobionts, in some instances, can be embedded in the commensal landscape and capable of blooming to cause infection in perturbed conditions (*7*,*8*). Because colonization can last for months to years, delineation between endogenous infection, in which a patient is infected with a pathogen from their own microbiota, and exogenous infection, in which they are infected with a pathogen more recently transmitted to them, is not always clear. Of note, pathogenic bacterial colonization in the intestine is normally limited by nonpathogenic commensal bacteria in the human microbiome, known as colonization resistance (*7*). Disruption of the intestinal microbial community with antibiotic prophylaxis before medical procedures or during treatment of unrelated syndromes reduces colonization resistance and opens the door for colonizing pathogens to cause infection, especially when the pathogen is resistant to the antibiotics causing the disruption.

**Table:** Key definitions used to describe decolonization and pathogen reduction to prevent antimicrobial resistance and healthcare-associated infections

Term	Definition
Colonization	Harboring living, actively dividing, and stable bacterial cell populations that do not cause symptoms of disease or infection.
Decolonization	Removing or reducing the burden of a pathogen, either temporarily or permanently.
Pathogen reduction	Substantial reduction of colonizing pathogen load, inclusive of, but not solely related to, decolonization, and more focused over a short period of increased infection or transmission risk.
Cross-transmission	Transmission of bacterial infection and antimicrobial resistance.
Opportunistic pathogen	Disease-causing microbes that can invade the body and cause disease under conditions of weakened immune defense.
Pathobiont	Opportunistic pathogenic bacteria that can emerge from the human microbiota to cause disease when its microbial ecology is disturbed.
Pathotype	A group of bacteria within the same species that can attack a host in different ways.

Nasal carriage of *Staphylococcus aureus* has long been considered a major risk factor for wound infections, surgical-site infections (SSIs), or bloodstream infections (BSIs) (*9*). *S. aureus* bacteremia has been shown to be caused by endogenous infections arising from a colonizing *S. aureus* strain; up to 80% of nosocomial bacteremia cases are linked to an endogenous source (*9*,*10*). In a large trial of >14,000 patients screened for *S. aureus* colonization, risk for nosocomial bacteremia was much higher for *S. aureus* nasal carriers than noncarriers (*10*). The HAI burden of *S. aureus* colonization is compounded by methicillin-resistant *S. aureus* (MRSA). MRSA has a higher attributable 30-day mortality risk than multidrug-resistant gram-negative bacteria (MDR-GNB) (*11*) and carries substantial risk for infection after discharge in colonized patients (*12*). One study estimated the pooled global prevalence of MRSA in elderly care centers to be >14%, illustrating the growing need for effective pathogen reduction and decolonization approaches for one of the most prevalent and threatening MDROs for global health (*13*).

In addition to MRSA colonization of the nares, unrecognized colonization with MDROs in the gastrointestinal tract and other body sites poses a risk in vulnerable patient populations (*14*). A recent systematic review and metaregression quantified the pooled cumulative incidence of infection in patients colonized with MDROs; intestinal colonization with MDR-GNB led to 14% incidence of infection at 30 days follow-up and vancomycin-resistant enterococci (VRE) led to 8% incidence (*15*). 

Intestinal colonization with extended-spectrum β-lactamase–producing Enterobacterales, a drug-resistant family of bacteria with limited treatment options, is associated with greatly increased incidence of BSIs (*16*). In a longitudinal study, increased relative abundance (i.e., >22%) of carbapenem-resistant Enterobacteriaceae in the microbiota of critically ill patients was associated with increased risk for BSIs (*4*). Likewise, hematopoietic stem cell transplantation patients who had >30% relative abundance of VRE in the microbiota were at a 9-fold higher risk for bloodstream VRE infections (*6*). Therefore, partially or completely reducing the colonizing load of a pathogen, especially during a period of critical illness or for high-risk patients, is a promising approach for HAI prevention.

## Evidence for and Current Applications of Pathogen Reduction

Pathogen reduction is central to some forms of currently recommended antibiotic prophylaxis backed by evidence-based guidelines from major public health organizations, specifically for prevention of SSIs (*17*). For example, use of combined oral antimicrobial prophylaxis before elective colorectal surgery and decolonization of surgical patients with antistaphylococcal agents for orthopedic and cardiothoracic procedures received high-quality evidence from a recent practice recommendation to prevent SSIs in acute-care hospitals (*18*). Both examples prevent infections by reducing potentially pathogenic bacterial bioburden and suppressing colonization. In our view, antibiotic selection for prophylaxis should match the expected susceptibility of colonizing pathogens; pathogen reduction in the form of prophylaxis comes at the expense of potentially increasing antimicrobial resistance. Although antibiotic prophylaxis before bowel surgery targets gram-negative and anaerobic bacteria from the gut, mupirocin applied to the anterior nares can decolonize or reduce local numbers of *S. aureus.*

To prevent HAIs more broadly, CDC guidance for preventing MRSA infections includes several core and supplemental strategies to implement decolonization and pathogen reduction, including intranasal mupirocin and chlorhexidine bathing (*19*). Evidence supporting that guidance includes the REDUCE MRSA cluster-randomized trial encompassing 74 intensive care units (ICUs) and nearly 75,000 patients (*20*). That trial reported a 37% reduction in MRSA-positive clinical cultures and a 44% reduction in BSIs from any pathogen by following universal decolonization with chlorhexidine bathing in routine ICU practice (*20*). The benefits of chlorhexidine for routine bathing were also evident after a cluster-randomized trial of 28 nursing homes in which universal decolonization with chlorhexidine and nasal povidone/iodine reduced prevalence of MDRO carriage and need for transfer to a hospital (*21*). An additional benefit of chlorhexidine bathing is a limited potential for unintended microbial consequences because this approach does not greatly disrupt the commensal skin microbiota (*22*). Given its broad-spectrum activity against gram-negative and gram-positive MDROs, chlorhexidine bathing represents an effective and microbiota-sparing pathogen reduction strategy to prevent HAI (*22*). Furthermore, pathogen reduction of the body surface is a widely available, noninvasive solution that, although in some instances challenging to implement, can be used at admission to healthcare settings to reduce general infection risk and increase patient safety facilitywide. The successes of both mupirocin and chlorhexidine as topical decolonization agents cannot be overstated in any discussion on pathogen reduction as a public health intervention for preventing HAIs. A comprehensive review and discussion of those decolonization strategies is available (*23*).

Despite its success, topical decolonization is not simple to implement, but pathogen reduction of the gastrointestinal tract can be even more challenging. One relatively well characterized method is selective decontamination of the digestive tract (SDD), practiced under guidelines in critically ill patients in the Netherlands (*24*). Application of SDD antibacterial suspensions along with short courses of intravenous third-generation cephalosporin has been associated with improved patient outcomes in critically ill hospital patients in the Netherlands, where AMR prevalence is relatively low (*24*). However, SDD effectiveness in settings of moderate to high AMR remains to be verified. The SDD strategy was shown effective at considerably reducing carbapenem-resistant *Klebsiella pneumoniae* in a cohort of colonized patients with severe underlying conditions, suggesting that a pathogen reduction approach might be suitable for critically ill patients colonized with *K. pneumoniae* (*25*). However, the combination of nonabsorbable and intravenous prophylactic antimicrobial drugs during SDD raises concerns for long-term effects on selecting for AMR in patients’ gut microbiota once SDD is discontinued (*26*). SDD of critically ill patients colonized with MDR-GNB has been most extensively explored in Europe, including a multiyear trial in Spain that reported marked reduction of MDR-GNB infections after SDD treatment in an ICU with high AR prevalence (*27*). Nevertheless, the European Committee of Infection Control has withheld recommendations for decolonization or pathogen reduction with SDD, citing major limitations in study heterogeneity, colonization pressure, and SDD agents (*28*).

Whether a universal approach to pathogen reduction is more effective than a targeted approach on the basis of screening patients for colonization with specific pathogens is still under scrutiny. Available evidence suggests a universal approach is more effective, for example, in reducing BSIs from any pathogen compared with targeted pathogen reduction of *S. aureus–*colonized patients only (*20*). The potential impact of effective pathogen reduction on illness, death, and costs is greatly influenced by additional indirect benefits that exponentially amplify the direct benefit to the index patient (*29*). Indirect benefits of pathogen reduction have the potential to extend beyond the patient to uncolonized persons through presumed decreases in pathogen shedding from recently decolonized patients, leading to a cascade of (theoretically) prevented infections and deaths (*29*). One cost-effectiveness analysis measured life-years gained after decolonizing antibiotic treatment compared with infection treatment only and described the cost-effective potential of decolonization in a long-term acute care hospital setting (*30*). Using a compartmental model, those analyses showed up to 40-fold more deaths prevented and up to 30-fold more BSIs prevented, and the cost effectiveness was reflected by negative incremental costs when indirect effects of decolonization were included (*30*). Thus, decolonization can improve not only individual patient safety but also plausibly that of an entire healthcare system by reducing the pathogen burden at the population level.

## Leveraging the Human Microbiome in Pathogen Reduction

In addition to the potential long-term AMR risks that could emerge from decolonization strategies that prioritize antibacterial prophylaxis, prophylaxis with broad-spectrum antibiotics including fluoroquinolones (e.g., in neutropenic cancer patients) carries high risk for antibiotic-associated adverse events, including *Clostridioides difficile* infection (CDI) (*31*). Therefore, alternative pathogen reduction approaches are needed. Pathogenic and often drug-resistant bacteria that increase and dominate the gut in clinical settings take advantage of ecologic disturbances in the microbiota during hospitalization. Intestinal domination with drug-resistant pathogens greatly increases risk for bacterial translocation in the gut, leading to bacteremia in vulnerable populations (*4*). To continue to elevate patient safety amid increasing AMR threats, future approaches should focus on microbiome-preserving or microbiome-restorative interventions that enrich beneficial populations of microbes to provide colonization resistance against pathogens. Thus, the functional roles of the human microbiome in colonization resistance should be considered for all decolonization strategies. The loss of microbial diversity in the intestine and the colonization resistance afforded by it resulting from antibiotic exposure, inflammation, or other perturbations, can lead to intestinal domination by pathobionts that produce new or emerging pathotypes (*7*).

Prior antibiotic use is a strong risk factor for healthcare-associated CDI (*31*). Asymptomatic *C. difficile* colonization is common in hospitalized patients and long-term care facility residents, but carriage might be transient depending on the stability of the microbiota. Microbiome disruption and immunosuppression increase CDI risk in colonized patients, and ≈10%–60% of patients colonized with toxigenic *C. difficile* develop symptomatic disease (*32*). High asymptomatic colonization rates of up to 18% in hospitals (*32*), up to 15% in long-term care facilities (*33*), and >50% in 1 reported outbreak setting (*33*) are alarming given the transmission potential of asymptomatic carriers (*32*,*33*). Although prophylactic oral vancomycin for preventing CDI in patients treated with systemic antibiotics is an active area of investigation, considerable long-term impacts of this strategy on the microbiome are possible (*5*). A stable gut microbiome serves as a primary defense from initial *C. difficile* colonization and prevents transition from colonization to symptomatic infection. Even though *C. difficile* colonization can exert toxigenicity at very low relative abundances in human microbiomes, more severe CDI symptoms are positively correlated with lower PCR cycle threshold values, which reflect higher pathogen loads (*34*). Thus, pathogen reduction could be an applicable approach for low abundance toxigenic gut colonizers.

A need for effective gut pathogen reduction strategies for *C. difficile* and other MDROs exists, as does a need for approved and standardized microbiome-based therapeutics aimed at prevention rather than treatment. Fecal microbiota transplantation (FMT) is one strategy to treat patients with recurring CDI who do not respond to standard therapies. A 2013 study reported a successful randomized control trial of FMT to treat recurring CDI (*35*). Since then, continued advancements in safety and efficacy of that treatment have been made through multiple clinical trials with largely favorable outcomes (*36*). Of note, in 2022, the US Food and Drug Administration (FDA) approved a fecal microbiota product under the trade name Rebyota (RBX2660; Ferring Pharmaceuticals, https://www.ferring.com) to treat recurring CDI after antibiotic therapy (*37*). That approval was followed by FDA approval of the oral product Vowst (SER-109; Seres Therapeutics, https://www.serestherapeutics.com) (*38*). Therefore, FMT and related live biotherapeutic products represent a promising microbiome-based therapy for recurring CDI. However, effective prevention and treatment of primary CDI and effective pathogen reduction for asymptomatic carriers are still needed. Challenges in regulation of FMT remain because of sample heterogeneity and other hurdles, but development of more standardized microbiome restoration products through defined bacterial consortia shows positive results after phase 2 clinical trials (*39*).

Fortifying the microbiome with live biotherapeutic products can influence and prevent disease by supplementing essential components of colonization resistance. One study pointed to post-FMT reductions in hospital stay duration and antibiotic use in addition to overall reduction of bacteremia and illness, despite modest long-term decolonization rates (*40*). FMT-treated recurring CDI patients also have been shown to have lower risk for BSI and reductions in hospitalizations compared with patients treated with antibiotics (*41*). Other clinical and immunologic outcomes that contribute to patient health and safety evidently are also contributing to the holistic benefits of pathogen reduction. 

The evidence for decolonization effectiveness and the importance of the human microbiome in colonization resistance still point to pathogen reduction as the primary or preferred mechanism for preventing infection. Another novel approach of preserving the microbiome is demonstrated by a placebo-controlled trial for *S. aureus* decolonization of healthy adults using a probiotic *Bacillus* spp. (i.e., a live biotherapeutic). Oral administration led to a 95% reduction in *S. aureus* total abundance from both the intestines and the nares without adverse effects or altering the microbiome (*42*). The use of live biotherapeutics in pathogen reduction should be further explored for potential clinical impact.

## Ideal Attributes of Current and Future Pathogen-Reducing Agents

To devise the best approaches, other ideal attributes of current and future pathogen-reducing agents should be considered (Figure). Given the central role of the microbiome in colonization resistance and the selective pressure that antimicrobial drugs can have on the human microbiota, selectivity is a crucial aspect of pathogen reducing agents, especially agents that directly kill or inhibit bacterial growth. Ideally selectivity would be limited to the pathogen or group in question, for instance, aerobic gram-negative spectrum used in SDD. In addition, the administration routes (e.g., topical chlorhexidine) or drug kinetic factors (e.g., nonabsorbable antibiotics) that limit distribution of the agent to the colonization site would protect the microbiota at other body sites. Chlorhexidine is one beneficial pathogen-reducing agent because it is an antiseptic agent that acts markedly differently from other current therapeutic antimicrobial agents. In addition, chlorhexidine has high potency for gram-positive organisms in relation to levels typically achieved when applied to the skin. 

**Figure:**
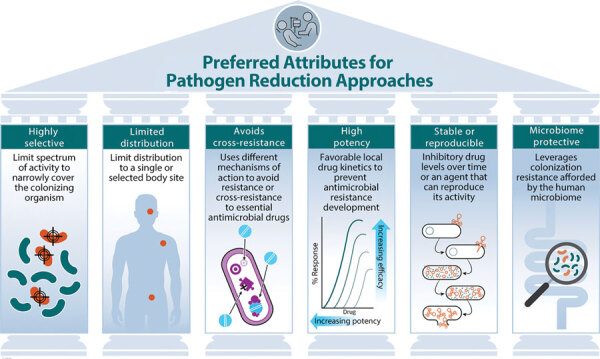
Preferred attributes for decolonization and pathogen reduction approaches to prevent antimicrobial resistance and healthcare-associated infections. Examples of these approaches include the following: highly selective, e.g., selective digestive decontamination targeting aerobic gram-negative bacilli; limited distribution, e.g., nonabsorbable antimicrobial drugs; avoids cross-resistance, e.g., chlorhexidine biocide; high potency, e.g., preventing selection of resistant mutations; stable or reproducible, e.g., use of phages to decolonize or reduce bacterial burden; and microbiome protective, e.g., using the human microbiome to spare beneficial microbes.

Another example of a potential future agent possessing several ideal attributes is lysostaphin, a bacteriocin with a limited spectrum of activity. Lysostaphin applied nasally is highly active in killing *S. aureus* relative to achievable concentrations (*43*).

Leveraging the microbiome is another ideal attribute of a pathogen-reducing agent. Future strategies that leverage the microbiome in pathogen reduction should expand applications beyond recurrent and primary CDI and capitalize on microbial ecology to achieve MDRO reduction while limiting use of antimicrobial agents. Results from PREMIX, a randomized controlled trial of FMT for MDRO decolonization, reported substantial reductions of colonization and infection, and replacement of extended-spectrum β-lactamase–producing *E. coli* strains by more susceptible strains after FMT (*44*). FMT for pathogen reduction in immunocompromised patients undergoing transplantation for hematologic malignancies is also effective for reconstituting the gut microbiota (*45*). 

Another preferred attribute is sustained pathogen reduction activity over time and the ability of an agent to replicate in the human microbiome or the environment. Pathogen reduction could revive interest in the use of bacteriophages, viruses that only infect bacteria, to target specific MDROs. One report highlights the development of an engineered phage combination, SNIPR001, used to reduce *E. coli* gut colonization by targeting *E. coli* strains that cause BSIs among hematology-oncology patients undergoing chemotherapy, similar to current fluoroquinolone prophylaxis (*46*). 

Microbiome-complementary therapies, such as FMT, and possible future use of bacteriophages for pathogen reduction represent novel interventions that promote antimicrobial stewardship along with patient well-being. Several ongoing registered clinical trials are using these pathogen reduction approaches and span various trial phases (Appendix Table).

## Pathogen Reduction Moving Forward

Pathogen reduction is an essential area for further development. Evidence suggests major benefits for reducing infection risk and improving associated clinical outcomes. Expanding the use of pathogen reduction approaches will require development of new diagnostic tests that can rapidly detect MDROs and quantify MDRO burden at certain anatomic sites. One novel approach applies an engineered reporter phage luminescence assay for swift and accurate point-of-care urinary tract infection diagnostics, which further doubles as rapid phage-patient matching for personalized therapy (*47*). New diagnostic testing will enable well-designed studies of novel agents that assess efficacy in reducing pathogen burden as well as clinical outcomes. A 2022 FDA-CDC public workshop addressed those issues (https://ftp.cdc.gov/pub/ARX-COMMUNICATIONS/pdf/CDC_FDA_Meeting). A primary driver of that workshop was the recognized need for drug development and registration pathways, at least for the regulatory framework in the United States. 

The approaches to pathogen reduction will involve drugs and biologics (e.g., live microbials, probiotics and prebiotics, and bacteriophages) that have vastly different regulatory bases and burdens of evidence. In some cases, such as with live microbials marketed as probiotics, avenues exist with current regulation and use as dietary supplements with allowable generalized health claims. Whether those health claims can cover pathogen reduction is unclear because the products are also intended for use in healthy populations and have different expectations than products used for pathogen reduction (*48*). Risks and challenges associated with treatment using live biotherapeutic products include the potential transmission of infectious agents (*49*), and the complex biologic interactions with host microbial communities, including the pharmacokinetics and pharmacodynamics associated with host effects on therapeutic product and vice versa (*50*). Likewise, interplay with microbiome succession and maturation, especially in specialized populations, such as infants, should be considered for analyses of risk in long-term colonizing products (*48*). Regardless, using an underlying mechanistic basis (whether drug or biologic), product development could benefit from focusing on attributes needed for established colonization in or on the human body to avoid the emergence of resistance. 

In conclusion, the approaches to pathogen reduction will clearly be multifaceted. Nonetheless, harnessing and applying our understanding of ecologic principles to address the pathogen burden in healthcare might promote enduring success in driving down infections while preserving the lifesaving utility of available therapeutic drugs.

Appendix Additional information on decolonization and pathogen reduction to prevent antimicrobial resistance and healthcare-associated infections.
