# Supplementary material for: Decolonization and Pathogen Reduction Approaches to Prevent Antimicrobial Resistance and Healthcare-Associated Infections
Source: Emerg Infect Dis. 2024 Jun;30(6):1069–76. doi: 10.3201/eid3006.231338 (PMC11138981; doi:10.3201/eid3006.231338)
Supplement: Appendix — Additional information on decolonization and pathogen reduction to prevent antimicrobial resistance and healthcare-associated infections. [file 23-1338-Techapp-s1.pdf]

Article DOI: https://doi.org/10.3201/eid3006.231338

EID cannot ensure accessibility for supplementary materials supplied by authors. Readers who have difficulty accessing supplementary content should contact the authors for assistance.

Decolonization and Pathogen Reduction Approaches to Prevent Antimicrobial Resistance and Healthcare-Associated Infections

Appendix

Appendix Table. Relevant ongoing registered studies investigating interventions for decolonization and pathogen reduction*

Clinical Trial	Phase	Pathogen reduction approach
Staph Household Intervention for Eradication (SHINE)	Phase 4	Personal and household environmental hygiene measures
Povidone-iodine to Stop Access-related Infections and Transmission of Staphylococcus Aureus (PAINTS)	Phase 4	Povidone-Iodine Topical Ointment
Decolonization of Carbapenem-resistant Enterobacterales (CRE) in Patients With Fecal Carriage of CRE With Neomycin	Phase 4	Neomycin
A Multi-center, Single-arm Trial Exploring the Safety and Clinical Effectiveness of RBX2660 Administered by Colonoscopy to Adults With Recurrent Clostridioides Difficile Infection (CDI-SCOPE)	Phase 3	Purified/processed fecal material
VE303 for Prevention of Recurrent Clostridioides Difficile Infection (RESTORATiVE303)	Phase 3	VE303 live biotherapeutic
ECOSPOR IV: An Open-Label Study Evaluating SER-109 in Recurrent Clostridioides Difficile Infection	Phase 3	Purified/processed fecal material
Decolonization of Gram-negative Multi-resistant Organisms (MDRO) With Donor Microbiota (FMT) (DEKODON)	Phase 3	Allogenic and autologous FMT
General Decolonization With Octenisan® Set Before Elective Orthopedic Surgery (BALGDEC)	Phase 3	Octenisan® wash lotion and Octenisan® nasal gel
Open-Label Extension of CP101 Trials Evaluating Oral Full-Spectrum Microbiota (CP101) in Subjects With Recurrence of Clostridium Difficile Infection (PRISM-EXT)	Phase 2	Purified/processed fecal material
Safety and Efficacy Study of VP20621 for Prevention of Recurrent Clostridium Difficile Infection	Phase 2	Single agent microbial therapeutic
Clinical Trial to Demonstrate the Effectiveness of Fecal Microbiota Transplantation for Selective Intestinal Decolonization of Patients Colonized by Carbapenemase-producing Klebsiella Pneumoniae (KAPEDIS)	Phase 2	Fecal microbiota transplantation
Safety and Efficacy of Oral Administration of the Phage Cocktail, VRELysin, In Healthy and VRE-Colonized Subjects	Phase 2	VRELysin phage cocktail
Cystic Fibrosis Bacteriophage Study at Yale (CYPHY)	Phase 2	YPT-01 phage therapy
Phage Therapy for the Treatment of Urinary Tract Infection	Phase 2	3-phage cocktail
A Multiple Dose Study to Evaluate Safety, Tolerability, PK, and Efficacy of SER-155 in Adults Undergoing HSCT	Phase 1	SER-155 defined consortium
A Study Investigating the Safety, Recovery, and Pharmacodynamics of Multiple Oral Administrations of SNIPR001 in Healthy Subjects	Phase 1	SNIPR001 engineered phage cocktail
MET-2 Clinical Study for Recurrent Clostridium Difficile Infection (CDI)	Phase 1	Defined consortium
Sentinel Cohort for the Response to Emerging Antimicrobial Resistance With Containment Microbiota Restoration Therapy Trial (REACT)	Phase 1	Allogeneic Microbiota in Glycerol

Efficacy and Safety of FMT for the Decolonization of MDROs in the Intestinal Tract	NA	Fecal microbiota transplantation
The Effect of Fecal Microbiota Transplantation on the Decolonization of Multidrug-resistant Organisms	NA	Fecal microbiota transplantation
Fecal Microbiota Transplantation (FMT) to Decolonize Antibiotic - Resistant Bacteria (ARB) - New Protocol (STOP-ARB2)	NA	Fecal microbiota transplantation

*Trials appear as listed on ClinicalTrials.gov, accessed March 18, 2024. Note that this list is not exhaustive because not all studies are required to be registered through this resource. NA, not applicable
